# Factors Influencing Risk for COVID-19 Exposure Among Young Adults Aged 18–23 Years — Winnebago County, Wisconsin, March–July 2020

**DOI:** 10.15585/mmwr.mm6941e2

**Published:** 2020-10-16

**Authors:** Rebecca F. Wilson, Andrea J. Sharma, Sarahjean Schluechtermann, Dustin W. Currie, Joan Mangan, Brian Kaplan, Kimberly Goffard, Julia Salomon, Sue Casteel, Ashley Mukasa, Niki Euhardy, Andrew Ruiz, Gregory Bautista, Erika Bailey, Ryan Westergaard, Douglas Gieryn

**Affiliations:** ^1^CDC COVID-19 Response Team, ^2^Winnebago County Public Health Department, Winnebago County, Wisconsin, ^3^Epidemic Intelligence Service, CDC; ^4^Bureau of Communicable Diseases, Division of Public Health, Wisconsin Department of Health Services.

On May 13, 2020, the Wisconsin Supreme Court declared the state’s Safer at Home Emergency Order (https://evers.wi.gov/Documents/COVID19/EMO28-SaferAtHome.pdf) “unlawful, invalid, and unenforceable,”* thereby increasing opportunities for social and business interactions. By mid-June, Winnebago County,^†^ Wisconsin experienced an increase in the number of infections with SARS-CoV-2, the virus that causes coronavirus disease 2019 (COVID-19), with the largest increase among persons aged 18–23 years (young adults) (*1*). This age group^§^ accounts for 12.5% of the population in the county. To identify factors that influence exposure to COVID-19 among young adults in Winnebago County, characteristics of COVID-19 cases and drivers of behaviors in this age group were examined. During March 1–July 18, 2020, 240 young adults received positive SARS-CoV-2 test results, accounting for 32% of all Winnebago County cases. In 30 key informant interviews, most interviewees reported exposure to misinformation, conflicting messages, or opposing views about the need for and effectiveness of masks. Thirteen young adults described social or peer pressure to not wear a mask and perceived severity of disease outcome for themselves as low but high for loved ones at risk. Having low perceived severity of disease outcome might partly explain why, when not in physical contact with loved ones at risk, young adults might attend social gatherings or not wear a mask (*2*). Exposure to misinformation and unclear messages has been identified as a driver of behavior during an outbreak (*3*,*4*), underscoring the importance of providing clear and consistent messages about the need for and effectiveness of masks. In addition, framing communication messages that amplify young adults’ responsibility to protect others and target perceived social or peer pressure to not adhere to public health guidance might persuade young adults to adhere to public health guidelines that prevent the spread of COVID-19.

SARS-CoV-2 spreads easily through person-to-person contact; certain behavioral factors (e.g., wearing masks, social distancing, and avoiding large gatherings) are effective in preventing COVID-19.^¶^ Young adults represent an increasingly large proportion of U.S. COVID-19 cases (*5*). A recent survey found that persons aged 18–24 years reported lower agreement with and adherence to public health guidance (e.g., wearing masks) compared with those aged ≥25 years (*2*). Identifying factors (e.g., perceived severity of disease outcome) that influence risk for exposure to COVID-19 and framing communication messages to target those factors might persuade young adults to engage in behaviors that are effective in preventing the spread of COVID-19 (*6*,*7*).

This study used a quantitative and qualitative approach to identify drivers of behavior that influence risk for exposure to COVID-19 among young adults. Characteristics (e.g., social gathering attendance, occupation, and age) of young adults with COVID-19 during March 1–July 18, 2020, and within Winnebago County, were obtained from Wisconsin’s Electronic Disease Surveillance System.** In addition, key informant interviews were conducted during July 9–22 with 30 persons, including 13 young adults, nine owners of business establishments employing and frequented by young adults (e.g., restaurants and bars), and eight community leaders^††^ (persons in various leadership roles) within Winnebago County using semistructured interview guides. Interviews did not knowingly include anyone who had received positive SARS-CoV-2 test results before the interview. Interview guides included questions to assess various factors (e.g., attitudes and perceptions).^§§^ Participants were recruited using snowball sampling, a method whereby enrolled participants refer other potential participants (*8*). Local health officials provided initial participant referrals. In-person interviews, lasting 30–75 minutes, were digitally recorded, transcribed, and analyzed using NVivo software (version 12; QSR International).^¶¶^ Analysis involved summarizing patterns of information shared by participants regarding their subjective experiences of the pandemic. All participants consented to being interviewed and received a gift card for participating.*** Interviews were conducted until thematic saturation was achieved and no new themes emerged.^†††^

By mid-June, after the Safer at Home Emergency Order was invalidated, Winnebago County experienced an increase in COVID-19 cases, with the largest increase among young adults (Figure). During March 1–July 18, 2020, young adults accounted for 240 (32%) of 757 cumulative COVID-19 cases in Winnebago County (Table 1). The majority of young adults were non-Hispanic White (72%); followed by other/unknown race/ethnicity (14%); Hispanic (7%); and non-Hispanic Black (4%). Over half were female (54%), and 72% reported being employed. Among those employed, 83% reported working outside of the home during their exposure period^§§§^; over half (58%) reported working outside of the home 2 days before symptom onset or positive specimen collection (i.e., during their contagious period^¶¶¶^). In addition, 38% reported attending a social gathering**** during their exposure period, and 84% reported clinical symptoms consistent with COVID-19.

**FIGURE:**
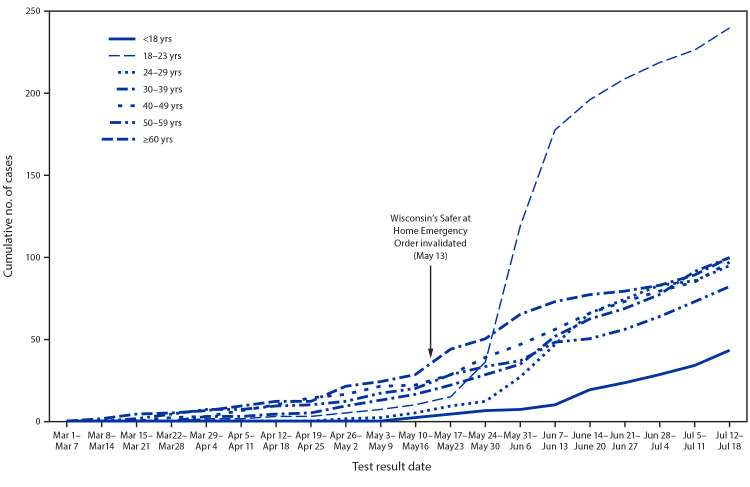
Cumulative number of confirmed COVID-19 cases, by age group (N = 757) — Winnebago County, Wisconsin, March 1–July 18, 2020 **Abbreviation:** COVID-19 = coronavirus disease 2019.

**TABLE 1:** Characteristics of confirmed cumulative COVID-19 cases among persons aged 18–23 years (N = 240), Wisconsin’s Electronic Disease Surveillance System — Winnebago County, Wisconsin,* March 1–July 18, 2020

Characteristic	No. (%^†^)
**Age, yrs**	
18	8 (3.3)
19	22 (9.2)
20	28 (11.7)
21	63 (26.3)
22	67 (27.9)
23	52 (21.7)
**Sex**	
Men	111 (46.3)
Women	129 (53.8)
**Race/Ethnicity**	
White, non-Hispanic	173 (72.1)
Hispanic	17 (7.1)
Black, non-Hispanic	10 (4.2)
Asian	3 (1.3)
American Indian	3 (1.3)
Other/Unknown	34 (14.2)
**Employment status/Occupation^§^**	
Employed	173 (72.1)
Restaurant/Bar	47 (19.6)
Health care	35 (14.6)
Other	91 (37.9)
Unemployed	41 (17.1)
Unknown	26 (10.8)
**Among employed (n = 173), worked 14 days before symptom onset or receiving positive test results (exposure period)**	
Yes	143 (82.7)
No	15 (8.7)
Unknown	15 (8.7)
**Among employed (n = 173), worked in the 2 days before symptom onset or positive specimen collection (contagious period)**	
Yes	101 (58.4)
No	37 (21.4)
Unknown	35 (20.2)
**Attended social gathering in the 14 days before symptom onset receiving positive test results^¶^**	
Yes	91 (37.9)
No	109 (45.4)
Unknown	40 (16.7)
Among those who reported attending a social gathering (n = 91), locations reported**	
House party	32 (35.2)
Domestic travel^††^	31 (34.1)
Restaurant or bar	30 (33.0)
Unknown location^§§^	14 (15.4)
**Symptoms**	
Symptomatic	202 (84.2)
Asymptomatic	38 (15.8)
Symptoms reported by respondents (n = 202)**	
Headache	117 (48.8)
Cough	106 (44.2)
Loss of taste or loss of smell	96 (40.0)
Fevers, chills, or night sweats	87 (36.3)
Sore throat or hoarseness	76 (31.7)
Runny nose, congestion, allergy, or sinus symptoms	73 (30.4)
Muscle aches	63 (26.3)
Fatigue, weakness, or dizziness	61 (25.4)
Nausea, vomiting, diarrhea, or abdominal pain	42 (17.5)
Shortness of breath, chest tightness, or chest pain	37 (15.4)

**Abbreviation:** COVID-19 = coronavirus disease 2019.

* COVID-19 cases in this report are specific to the Winnebago County Health Department jurisdiction and do not include COVID-19 cases that fall within the City of Menasha Health Department jurisdiction and the City of Appleton Health Department jurisdictions.

^†^ Percentages might not sum to 100% because of rounding.

^§^ Five young adults reported employment at more than one employer but were counted only once under restaurant/bar (two) or health care (three). Young adults who reported their employment status as employed and student are counted under employed. Young adults who reported their occupation as student and did not include any additional information about occupation type are counted under unemployed.

^¶^ Social gathering refers to the COVID-19 patient reporting attending a gathering, party, or meeting with people from outside of their household in the 14 days before symptom onset or receiving positive test results. Wisconsin’s Electronic Disease Surveillance System does not provide a minimum number of participants to qualify as a social gathering.

**Characteristic is not mutually exclusive.

^††^ Domestic travel is categorized as a social gathering if the COVID-19 patient reported making a journey, out of town, to attend a gathering, party, or meeting with people from outside of their household in the 14 days before symptom onset or receiving positive test results.

^§§^ Unknown location reflects COVID-19 positive patients who reported attending a social gathering in the 14 days before symptom onset or receiving positive test results but did not report the location of the social gathering.

Among the 13 young adults interviewed, nine were women, all were employed, and all were either enrolled in college or had graduated from college within the last year. Common themes that emerged during interviews as drivers of behavior were social or peer pressure, social interactions, attitudes regarding public health guidance, perceived severity of disease outcome, perceived responsibility to others, workplace COVID-19 mitigation measures, absence of countywide measures, identifying a trusted source for COVID-19 information, and exposure to misinformation, conflicting messages, or opposing views regarding masks (Table 2). In the analysis of interviews, young adults described feeling social or peer pressure to not wear a mask, reportedly receiving “negative reactions” or “odd looks” from others when wearing a mask, or feeling “weird” about wearing a mask. Young adults reported limiting social interactions; however, many reported engaging in social activities (e.g., attending a bonfire or bar) that exposed them to multiple persons. Young adults reported wearing masks when shopping, most held favorable views of public health guidance (e.g., wearing masks), and a few had negative or questioning views of masks and social distancing. Most young adults indicated they would likely be asymptomatic or have mild or flu-like symptoms if they were to receive a positive test result or “had peers who had tested positive and those peers hardly even had symptoms.” Young adults reported having loved ones at risk for severe COVID-19–associated outcomes and expressed a sense of responsibility to those loved ones and the broader community. Moreover, most young adults voiced concerns about exposure to SARS-CoV-2 within their workplaces and reported exposure to misinformation, conflicting messages, or opposing views regarding the need for or effectiveness of masks.

**TABLE 2:** Themes from key informant interviews with young adults aged 18–23 years (n = 13), business owners* (n = 9), and community leaders^†^ (n = 8) — Winnebago County, Wisconsin, July 9–22, 2020

Theme	Example quotes
**Young adults**	
Social or peer pressure	“I felt like everybody else in here is not going to wear a mask, I might as well just go in there and not wear a mask as well. I don't want to be seen as different.”
	“When you're at your friend's, you don't want to be ‘that’ person that wears the mask, because then you look like a weirdo, you know.”
	“So, like for me seeing everyone not wearing masks and me being the only one, I'm like yeah, I feel pressured to take it off, and I don't want that, so I'll leave.”
Social interactions	“I've chosen to eat outside. I've chosen to do the things that I think are good that I also like to do. I felt like that was a risk versus a reward type of thing.”
	“[My friends and I] don’t wear masks together, but whenever I go out with them, we always just go to an outdoors place because we’re not in a bar or restaurant or anything like that. If you limit the amount of people you see and your friends also do the same, I feel comfortable.”
Attitudes regarding public health guidelines (e.g., wearing masks and social distancing)	“I personally feel like masks are a very effective way to stop the virus spread or at least control it.”
	“The isolation and the masks and everything, I just don't know that that's really necessary……Like I said, I'm not a scientist. I don't know. I'm questioning it. It's a little scary to me. Because if this is something that they're mandating, like what else is going to come next?” “But I just–like, that gets into personal beliefs.”
Perceived severity of disease outcome	“I know like five people that have had COVID, and they're all fine. I don't know anybody that's died and some of them have hardly even had symptoms.”
	“I hear most of it, you're probably like asymptomatic. I don't want to speak on it and jinx myself. So, I probably wouldn't show many signs [if I tested positive for COVID-19].”
Perceived responsibility to others	“For me it's more of who am I affecting the most. When it comes to, like, my grandparents or people at the grocery store, I don't want–even if do have it, and if I don't have any symptoms, why spread it to other people?”
	“I'm most worried about giving it to my dad. He's not in great health.”
Workplace COVID-19 mitigation measures	“I feel like if I went to my manager and asked him if we could do more, he would not take anything well, or he wouldn't implement anything. So, that's frustrating.”
	“We are actually not [required to wear masks at work], which is weird, in my personal opinion, but we are being very safe about it.”
Exposure to misinformation, conflicting messages, or opposing views regarding public health guidance^§^	“I think it's just hard, because nobody has the same message, and I feel like since it's a pandemic, and since it's a health issue, it shouldn't be about confusing messages. I think because it's confusing, that's makes me not really want to listen to anything.”
	“Some people are saying we need to wear masks for public health. Some people are saying they don’t work… So, it’s super hard to trust…”
	“I think definitely looking at [local and national leaders] and just seeing them not wear a mask. I think that has a really big effect on people and their own perception of the virus.”
Business owners*	
Lack of countywide measures	“They should mandate masks right this second. They should have done it two weeks ago, and the pushback was terrible.”
	“I would say the main thing is, that without a [county-wide] mandate for [masks] and knowing that many of my competitors are just not going to [require masks], that is my biggest barrier to [requiring masks].”
	“If I said, ‘you guys have to wear a mask,’ they'd walk down to the next bar that's not requiring a mask. I can guarantee that. It's competition, and it's a competition.”
Trusted source for COVID-19 information	“My main thing is I get that email every day from the [local health department], and that's where I go [for information on COVID-19].”
	“Within the county health department, their dashboards are great on a daily basis…..to understand daily where we are as a snapshot.”
Exposure to misinformation, conflicting messages, or opposing views regarding public health guidance^§^	“There are people who don't think [COVID-19] is real and that it doesn't exist, and there are people who think that wearing a mask impedes in their freedom and telling people where to sit [6 ft apart] impedes on their freedom as well, and they will not follow it regardless.”
	“We don't have any leadership from the top. You get these mixed signals. Who do I trust?”
**Community leaders^†^**	
Exposure to misinformation, conflicting messages, or opposing views regarding public health guidance^§^	“And it's, it's just been a disaster from a PR perspective for getting good information, accurate information out……. In the meantime, we're all bad people you know because we're not adhering to whatever they want us to adhere to.”
	“When you have [professionals] that don't think it's a good idea to self-quarantine, an ordinary person is going to sit there and say “well, [they] must know better.”
Perceived severity of the pandemic	“They [federal, state, and local public health agencies] have all done a crappy job of selling why this is bad, and that's why nobody believes it.”
	“I might not call it a pandemic, but until the numbers get higher than the regular flu, in my mind it's still a nasty flu.”
Trusted source for COVID-19 information	“[The local county health department] has done a good job with visibility, I believe.”
	“[We’re] being asked to wear a mask and do all sorts of things, you know. And I'm saying it's being based on wrong information, [bad data].”

**Abbreviation:** COVID-19 = coronavirus disease 2019.

* Business owners are owners of establishments employing and frequented by young adults (e.g., restaurants and bars).

^†^ Community leaders were interviewed to gain an understanding of broader concerns related to COVID-19 and its impact within the community, but because of their diverse roles within the community, results from those interviews were not analyzed for themes but presented as salient concerns raised by community leaders.

^§^ Exposure to misinformation, conflicting messages, or opposing views regarding public health guidance was reported within all interviewee groups. To facilitate interpretation and analysis of this theme, these three salient issues were reported under one theme because of their similarities.

Among interviewed business owners (nine) and community leaders (eight), all business owners identified local health officials as trusted sources for COVID-19 information, yet a few community leaders did not. Further, many business owners and community leaders reported exposure to misinformation, conflicting messages, or opposing views regarding the need for or effectiveness of masks. Business owners indicated they had implemented some control measures (e.g., hand-hygiene stations and mask-wearing); however, many reported discontinuing mask-wearing requirements for reasons such as not wanting to offend customers or perceived competition with similar establishments. Business owners perceived the absence of a countywide mask ordinance as a barrier to reimplementing mask-wearing requirements within their establishments and some spontaneously indicated that if a mask ordinance was implemented, they would comply.

## Discussion

Wisconsin’s Electronic Disease Surveillance System data indicated social interactions and workplace and community transmission likely contributed to the spread of COVID-19 among young adults in Winnebago County. Nearly three quarters (72%) of young adults with COVID-19 were employed, and over one half (58%) worked outside of the home while contagious, increasing the risk for transmitting SARS-CoV-2 to the broader community. Among young adult interviewees with jobs that entailed interaction with the public, many voiced concerns about workplace exposure, underscoring the importance of businesses implementing control measures (e.g., requiring masks) consistent with published guidance,^††††^ especially when physical distancing is difficult. These concerns, coupled with the fact that most business owners identified the absence of a countywide mask ordinance as a barrier to reimplementing mask-wearing requirements within their establishments, highlight the benefits that might come from implementing a countywide mask ordinance (*9*). Given that business owners and most community leaders trusted local health officials for COVID-19 information, businesses could collaborate with local health officials in implementing control measures tailored to their needs. Among the few community leaders who distrusted COVID-19 information shared by local health officials, that distrust appeared to stem from exposure to misinformation and conflicting messages regarding the severity of the pandemic, which in turn seemed to influence their views about the extremeness of broader community mitigation measures (e.g., the Safer at Home Order). Lack of trust can influence adherence to public health guidance.^§§§§^

Some young adults admitted to not wearing a mask when socializing with friends, which might indicate a sense of security when interacting with friends. Moreover, the expectation that they would likely be fine if they contracted COVID-19, coupled with social or peer pressure, might help explain transmission patterns among young adults. Although young adults perceived a low severity of disease outcome for themselves, many expressed concerns about transmitting SARS-CoV-2 to loved ones at risk and to the broader community. Having a sense of responsibility to others might explain why young adults reported wearing masks when shopping and why most held positive views of masks. However, when not in physical contact with loved ones at risk, young adults might choose to not wear a mask or to attend larger gatherings with peers who might also perceive a low severity of disease outcome for themselves. Exposure to misinformation and conflicting messages regarding masks might make it difficult to know what information to trust, underscoring the importance of providing clear and consistent messages during an outbreak (*3*,*4*). Among the few young adults who expressed negative attitudes about masks and social distancing or who had questions about the effectiveness of masks, those views appeared to be based on the expressed need to make their own choices (i.e., personal agency).

The findings in this report are subject to at least four limitations. First, interviews were conducted in Winnebago County; therefore, findings are not widely generalizable. Second, self-reported information collected in Wisconsin’s Electronic Disease Surveillance System and from interviews is subject to social desirability bias and might have led to underestimations of some characteristics and factors. Third, interviewees identified through snowball sampling might have similar characteristics; thus, this report might not capture representativeness of diverse responses. Finally, missing information in text fields could have led to underestimations of some characteristics.

Despite limitations, this report provides a framework for tailoring communication messages that are empathetic, that amplify personal responsibility and responsibility to protect others, and that focus on perceived pressure to not wear a mask, all of which might persuade young adults to adhere to public health guidelines (e.g., wearing masks) that prevent the spread of COVID-19. Masks are an effective tool to prevent the spread of COVID-19 (*9*), and current CDC guidance recommends universal masking to prevent SARS-CoV-2 transmission.^¶¶¶¶^ This report further underscores the importance of providing clear and consistent messages regarding need for and effectiveness of masks, because consistent messages could help increase widespread adoption of evidence-based guidance (*3*).

**Summary**

**What is already known about this topic?**

Young adults represent an increasingly large proportion of U.S. COVID-19 cases.

**What is added by this report?**

In Winnebago County, Wisconsin, perceived low severity of disease outcome; perceived responsibility to others; peer pressure; and exposure to misinformation, conflicting messages, or opposing views regarding masks were identified as drivers of behaviors that might influence risk for COVID-19 exposure among young adults.

**What are the implications for public health practice?**

Identifying factors that influence risk for COVID-19 exposure and framing messaging to target those factors could help persuade young adults to adhere to public health guidelines that prevent the spread of COVID-19. Providing clear and consistent messages regarding the need for and effectiveness of masks could help increase widespread adoption of evidence-based guidance.

## Footnotes

*
https://www.wicourts.gov/sc/opinion/DisplayDocument.pdf?content=pdf&seqNo=260868 .

† The COVID-19 data provided in this report are specific to the Winnebago County Health Department (WCHD) jurisdiction. Data for the portions of the City of Menasha or City of Appleton that fall within Winnebago County are not included. Data provided on the Wisconsin Department of Health Services website might be different than the information provided by WCHD because the state reports data for the entire county, which includes those portions of Menasha and Appleton.

§ Percentage of population includes persons aged 18–24 years in Winnebago County, Wisconsin, and were obtained using the U.S. Census 2018 American Community Survey data. Population data for young adults aged 18–23 years were not available for this report. https://data.census.gov/cedsci/table?g = 0500000US55139.060000&y = 2018&d = ACS%205-Year%20Estimates%20Detailed%20Tables&tid = ACSDT5Y2018.B01001.

¶
https://www.cdc.gov/coronavirus/2019-ncov/prevent-getting-sick/how-covid-spreads.html .

** In Wisconsin’s Electronic Disease Surveillance System, information for occupation and social gathering is systematically collected in various formats (e.g., text field and drop-down menu options); thus, abstraction of free text fields was completed to enhance data completeness and accuracy of these variables.

†† Community leaders were interviewed to gain an understanding of broader concerns related to COVID-19 and its impact within the community, but because of their diverse roles within the community, results from those interviews were not analyzed for themes but presented as salient concerns raised by community leaders. Thus, NVivo software was not used to analyze interviews for community leaders. Broader concerns expressed by community leaders that paralleled those of business owners and young adults were presented along with themes that emerged from interviews with business owners and or young adults.

§§ The interview guide for young adults included a range of questions and were abbreviated for this report to include questions regarding social interactions with peers, adherence to and attitudes about public health guidance, perceived severity of disease outcome, perceived responsibility to others, and social or peer pressure. The interview guide for business owners included a range of questions and were abbreviated for this report to include questions regarding trusted sources of COVID-19 information, main concerns regarding the pandemic, and barriers to implementing public health guidance within their establishments. The interview guide for community leaders included questions regarding participants' appraisal of the pandemic, main concerns regarding the pandemic, and trusted sources of COVID-19 information.

¶¶ https://www.qsrinternational.com/nvivo-qualitative-data-analysis-software/home.

*** Providing incentives to participants have been found to increase participation in studies (https://link.springer.com/article/10.1023/A:1025023600517). Thus, in this study, a gift card in the sum of $25 was provided to participants, and the amount was based on the need to balance motivating interviewees to participate without offering a coercive sum (i.e., a sum that a low-income individual would find difficult to refuse). Two interviewees declined the offer of a gift card.

††† Thematic saturation, which is often used to determine sample size in qualitative data collection (e.g., key informant interviews), is achieved when no new information or salient themes arise from data collection. Probing, in-depth responses provided by interviewees, and the number of salient issues being discussed are some factors that influence when thematic saturation is reached. https://www.ncbi.nlm.nih.gov/pmc/articles/PMC6010234/.

§§§ Exposure period is defined as the 14 days preceding symptom onset or receiving positive test results.

¶¶¶ Contagious period is defined as working outside of the home the 2 days before symptom onset or positive specimen collection. Information for worked during contagious period was determined by examining information contained in the Facility Intervention section and the date of onset of symptoms, which are both systematically collected in Wisconsin’s Electronic Disease Surveillance System. Abstraction of free text fields was completed to enhance data completeness and accuracy of data collected on patients reporting working outside of the home in the 2 days before symptom onset or positive specimen collection contagious period.

**** Social gathering refers to the COVID-19 patient reporting attending a gathering, party, or meeting with people from outside of their household in the 14 days before symptom onset or receiving positive test results. Wisconsin’s Electronic Disease Surveillance System does not provide a minimum number of participants to qualify as a social gathering.

††††
https://www.cdc.gov/coronavirus/2019-ncov/community/organizations/business-employers/bars-restaurants.html#anchor_1589927161215 .

§§§§
https://www.cdc.gov/eis/field-epi-manual/chapters/Communicating-Investigation.html .

¶¶¶¶ https://www.cdc.gov/media/releases/2020/p0714-americans-to-wear-masks.html.
